# Glycoluril–tetrathiafulvalene molecular clips: on the influence of electronic and spatial properties for binding neutral accepting guests

**DOI:** 10.3762/bjoc.11.115

**Published:** 2015-06-17

**Authors:** Yoann Cotelle, Marie Hardouin-Lerouge, Stéphanie Legoupy, Olivier Alévêque, Eric Levillain, Piétrick Hudhomme

**Affiliations:** 1 Université d'Angers, Laboratoire MOLTECH-Anjou, CNRS UMR 6200, 2 Boulevard Lavoisier, F-49045 Angers, France

**Keywords:** donor–acceptor interactions, glycoluril, molecular clips, supramolecular chemistry, tetrathiafulvalene

## Abstract

Glycoluril-based molecular clips incorporating tetrathiafulvalene (TTF) sidewalls have been synthesized through different strategies with the aim of investigating the effect of electrochemical and spatial properties for binding neutral accepting guests. We have in particular focused our study on the spacer extension in order to tune the intramolecular TTF···TTF distance within the clip and, consequently, the redox behavior of the receptor. Carried out at different concentrations in solution, electrochemical and spectroelectrochemical experiments provide evidence of mixed-valence and/or π-dimer intermolecular interactions between TTF units from two closed clips. The stepwise oxidation of each molecular clip involves an electrochemical mechanism with three one-electron processes and two charge-coupled chemical reactions, a scheme which is supported by electrochemical simulations. The fine-tunable π-donating ability of the TTF units and the cavity size allow to control binding interaction towards a strong electron acceptor such as tetrafluorotetracyanoquinodimethane (F_4_-TCNQ) or a weaker electron acceptor such as 1,3-dinitrobenzene (*m*-DNB).

## Introduction

Thanks to its remarkable redox properties and strong π-donating character demonstrated with the pioneering work of F. Wudl in the early 1970s [1], tetrathiafulvalene (TTF) has become one of the most popular electroactive frameworks used in materials science [2–5]. In addition to the well-known access to molecular conductors [6–7] and TTF-acceptor assemblies [8–9], new concepts have been explored in the field of supramolecular chemistry using the TTF unit as a powerful electroactive building block [10]. Exploiting its peculiar electronic (two successive reversible oxidation steps giving rise to three stable redox states) characteristics, more and more sophisticated TTF-based supramolecular systems have been designed, being able to operate as machines, chemical sensors, redox-switchable ligands, molecular shuttles, molecular switches and logic gates [11]. Considering that molecular receptors prone to specifically recognize neutral molecules through donor–accceptor interactions are of particular interest [12], the unique π-donating ability and the planar geometry of the TTF building-block are suited to the construction of such receptors. Nevertheless, the incorporation of TTF as a π-donating element in molecular clips or tweezers for recognition of neutral electron acceptor guests is still relatively unexplored [13].

Molecular clips and tweezers can be defined as receptors presenting an open cavity composed of two interaction sites which are separated by a spacer [13–15]. Ideally, these systems are designed with the aim of sandwiching a molecular guest with a high degree of selectivity and control of the interactions between the guest and the sidewalls. While the nature and the flexibility of the spacer between the two sidewalls play a critical role in the recognition process, we were interested in rigidifying the molecular clips in order to favor the “lock and key” model [15]. This rigid structure offers the possibility to orient the interaction sites in a defined manner allowing an increase of the binding strength with the guest. In the last two decades, glycoluril-based molecular clips have shown that they were capable of acting as excellent receptors by exploiting the distance between the two aromatic sidewalls which is usually close to 7 Å. Since then, a large number of host systems based on this principle have been synthesized in order to use them for recognition of aromatic guest molecules through π–π interactions [16–17].

**Figure 1:**
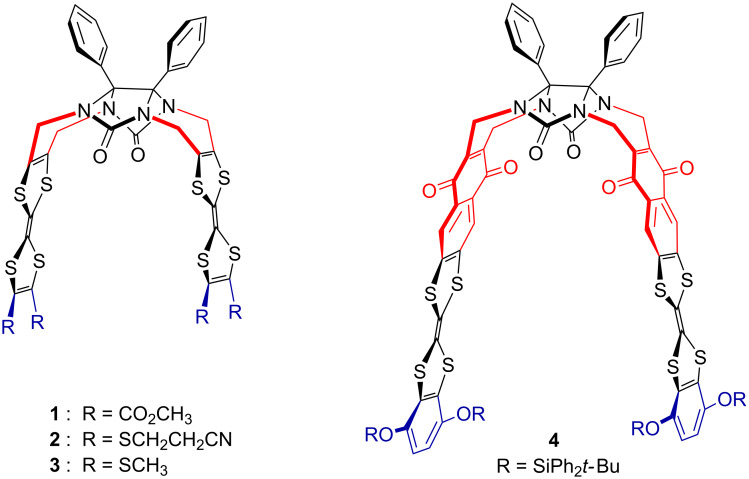
Structures of molecular clips **1–4**.

We recently described various glycoluril-based molecular clips **1**–**4** [18–19] (Figure 1) for which the structure varies by: i) the nature of connection between the glycoluril spacer and the TTF sidewalls, ii) the nature of the peripheral substituents on both TTF pincers. In this full research paper we propose to study how these structural parameters influence the electron donating ability of TTF pincers and the size of the cavity and how the control over the inter-TTFs distance within the molecular clip impacts the ability for sandwiching neutral acceptor guests. Those studies are supported by electrochemical analyses (including simulation), time-resolved spectroelectrochemical experiments and UV–visible titrations.

## Results and Discussion

### Synthesis

**Scheme 1:**
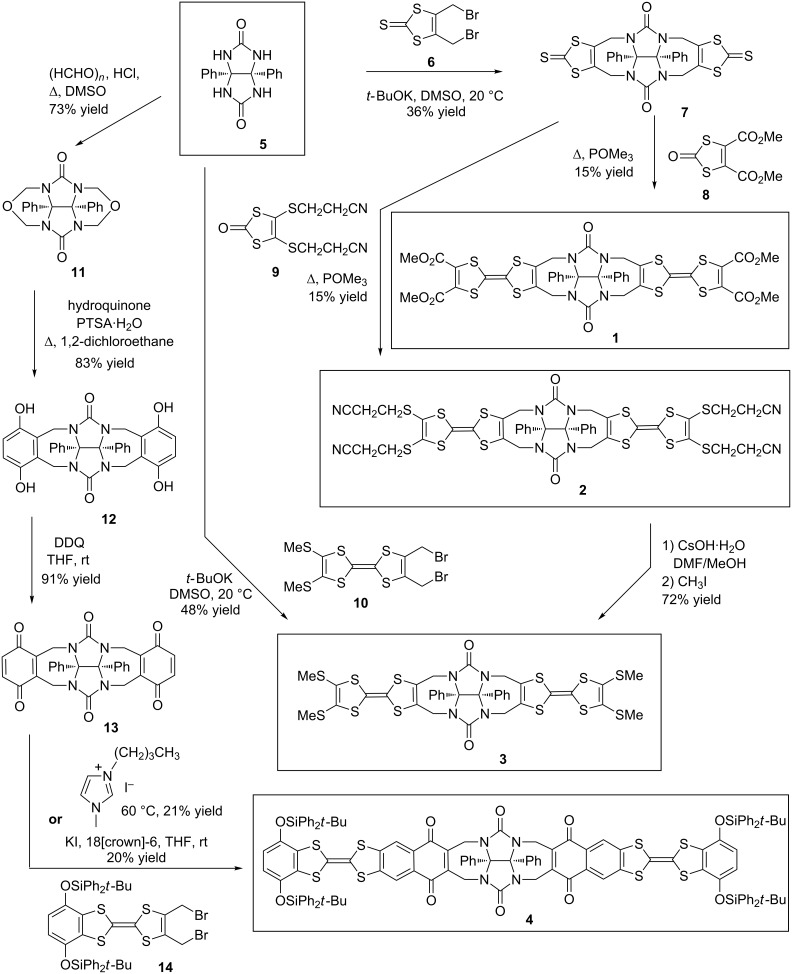
Different routes developed for the synthesis of molecular clips **1–4**.

We have designed molecular clips **1–4** through three different synthetic strategies starting from diphenylglycoluril **5** (Scheme 1). The key building-block **5** is available in multigram scale by reaction of benzil with urea [20]. The first approach leading to molecular clips **1** and **2** is based on a straightforward double nucleophilic substitution leading to a seven-membered ring using 4,5-bis(bromomethyl)-2-thioxo-1,3-dithiole (**6**) [21].

**Scheme 2:**
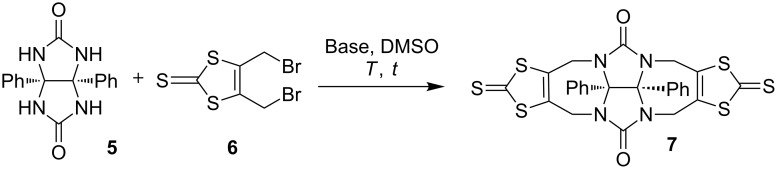
Reaction between diphenylglycoluril with 4,5-bis(bromomethyl)-2-thioxo-1,3-dithiole.

In order to determine the optimized experimental procedure to carry out the urea N-alkylation of compound **5**, we took advantage from literature of previous works realized on glycoluril for such reaction using a 2,3-bis(halogenomethyl)aryl derivative. Reported procedures were using KOH [22–23], *t-*BuOK [24–26] or NaH [27] as the base with DMSO as an aprotic polar solvent. Different experimental conditions were tested for the reaction between diphenylglycoluril **5** and 4,5-bis(bromomethyl)-2-thioxo-1,3-dithiole (**6**) as the electrophilic reagent (Scheme 2) by varying parameters such as the nature of the base, the reaction time and the temperature (Table 1). Optimal conditions were using four equivalents of *t-*BuOK in anhydrous DMSO by keeping the temperature at 20 °C with a cryostat apparatus for 3 h. Compound **7** was isolated in 36% yield after purification by silica gel chromatography. We should note that an increase of temperature resulted in the degradation of starting material **6**.

**Table 1:** Optimized experimental conditions for the synthesis of compound **7**.

Base	Temperature (*T*)	Reaction time (*t*)	Yield [%]

KOH	rt	24 h	6
NaH	rt	24 h	5
KOH	rt	4 h	12
*t-*BuOK	rt	4 h	19
*t-*BuOK	Controlled 30 °C	4 h	12
*t-*BuOK	Controlled 20 °C	3 h	36

This first strategy to reach clips **1** and **2** was considering the trimethylphosphite-mediated cross-coupling reaction involving 2-oxo-1,3-dithiole moiety **8** [28] as an efficient route to prepare dissymmetrical TTF derivatives [29]. The polarity of compounds which were formed was sufficiently different to allow an efficient purification by silica gel chromatography affording molecular clip **1** in 15% yield. This route was successfully applied using compound **9** [30] and clip **2** was also isolated in 15% yield. Target clip **3** was finally obtained in 72% yield by deprotection of precursor **2** using CsOH·H_2_O in DMF/MeOH mixture [31], followed by the tetraalkylation of tetrathiolate with iodomethane. Considering that this multi-step synthesis was affording molecular clip **3** in an overall 4% yield starting from diphenylglycoluril **5**, we took advantage of the accessibility of 2,3-bis(bromomethyl)TTF **10** [32] bearing methylsulfanyl groups to investigate a more straightforward strategy. Alternatively, the above methodology described for the preparation of compound **7** was efficiently applied to reach molecular clip **2** in 48% yield using similar experimental conditions.

Whereas the synthesis of molecular clips **1**, **2** and **3** uses the nucleophilic substitution onto diphenylglycoluril **5** as a key-step, the introduction of the naphthoquinone spacer in clip **4** required the previous transformation of compound **5** into a glycoluril-based framework possessing quinone moieties for developing a Diels–Alder cycloaddition strategy. Thus compound **12** [33] was prepared in 73% yield by treatment with an excess of hydroquinone in 1,2-dichloroethane using a Friedel–Crafts alkylation as an electrophilic aromatic substitution onto compound **11** [34]. The hydroquinone moieties were subjected to a dehydrogenation reaction using DDQ in THF to reach desired glycolurildiquinone **13** [35] in 91% yield. The Diels–Alder cycloaddition was carried out by treatment of bis-dienophile **13** with TTF derivative **14** [36], able to give rise in situ to the transient diene by reductive elimination using naked iodide [37–39] or the iodo-ionic liquid 1-butyl-3-methylimidazolium iodide [40]. After purification by column chromatography on silica gel, we noted that complete aromatization has occurred concomitantly and molecular clip **4** was isolated in around 20% yield.

**Figure 2:**
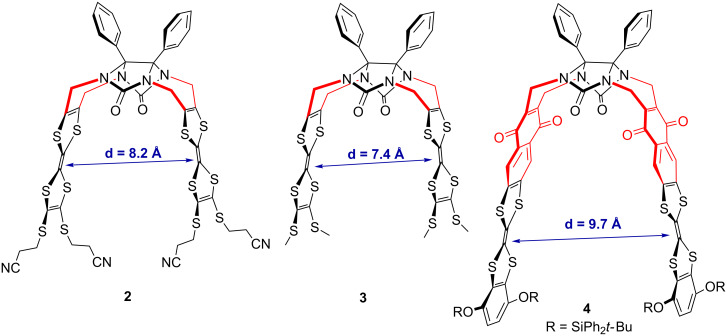
Intramolecular distances between TTF moieties from X-ray analysis for clips **2** and **3** and theoretical calculations for clip **4**.

Whereas all attempts at growing crystals of molecular clips **1** and **4** of sufficient quality for X-ray analysis have been unsuccessful so far, suitable single crystals were obtained by slow diffusion from a solution of clips **2** and **3** in a CH_2_Cl_2_/hexane mixture. Considering the central double bond of each TTF moiety, the intramolecular wall-to-wall distance was determined to be equal to 8.25 Å and 7.41 Å for clip **2** and **3**, respectively (Figure 2) [18]. Then the corresponding wall-to-wall distance between the two TTF units was determined by theoretical calculations using the semi-empirical AM1 method in the case of **4** [19]. This distance was estimated to be equal to 9.7 Å indicating that this enlargement around 2 Å was the result mainly of the spatial contribution of the additional naphthoquinone spacer in molecular clip **4**.

### Electrochemical properties

The electrochemical behaviour of molecular clips **1–4** were determined using cyclic voltammetry experiments (Table 2).

**Table 2:** Apparent redox potentials of molecular clips **1, 2, 3** and **4** reported vs Fc^+^/Fc in 0.1 M TBAPF_6_/CH_2_Cl_2_/CH_3_CN 3:1 on glassy carbon electrode at 100 mV·s^−1^. Absolute errors on potentials were found to be around +/−10 mV.

Clip	*E*_app_^0^_red1_	*E*_app_^0^_ox1_	*E*_app_^0^_ox1’_	*E*_app_^0^_ox2_

**1**	–	+0.06	+0.21	+0.51
**2**	–	+0.03	+0.14	+0.45
**3**	–	−0.05	+0.06	+0.36
**4**	−1.05	+0.28		+0.65

**Figure 3:**
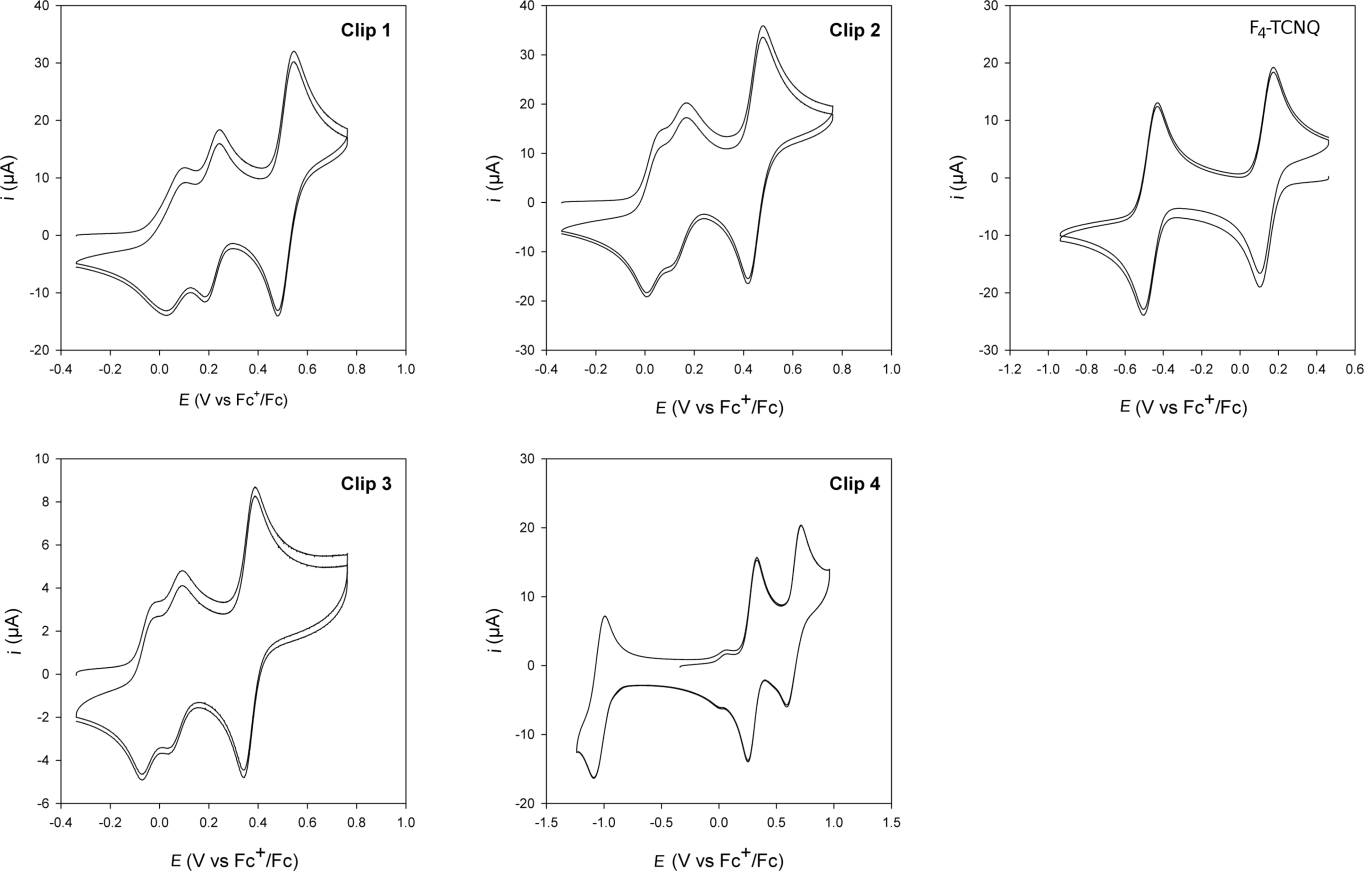
Cyclic voltammograms of molecular clips **1**, **2**, **3**, **4** and F_4_-TCNQ at 10^−3^ M in 0.1 M TBAPF_6_/CH_2_Cl_2_/CH_3_CN 3:1 on a glassy carbon electrode at 100 mV·s^−1^.

These studies displayed a different electrochemical behavior for clips **1**, **2** and **3** in comparison to clip **4**. In the latter case, the cyclic voltammogram (CV) showed two oxidation waves at *E*_app_^0^_ox1_ = +0.28 V and *E*_app_^0^_ox2_ = +0.65 V vs Fc^+^/Fc corresponding to the successive generation of cation radical then dication species simultaneously on each TTF framework (Figure 3). This unique first two-electron oxidation step indicates that the two TTF units are electrochemically equivalent, thus excluding the presence of intra- or intermolecular electronic interactions between them. It should be noted that the electroactive naphthoquinone acceptor group incorporated in the spacer unit is characterized by a reduction wave (*E*_app_^0^_red1_) at −1.05 V vs Fc^+^/Fc. On the contrary, the CV of clips **1**–**3** shows a significant splitting of the first oxidation wave (*E*_app_^0^_ox1_ and *E*_app_^0^_ox1’_), suggesting the presence of intermolecular (between two clips) or intramolecular (within a clip) interactions between two TTF units (i.e., mixed valence and/or π-dimer) [41]. Eventually, the reversible two-electron process (i.e., 1 e^−^ on each TTF^+·^ unit) for the second oxidation step (*E*_app_^0^_ox2_) leading to fully oxidized TTF units is in accordance with independent TTF^2+^ states subject to repulsive electrostatic interactions. Such splitting phenomenon of the first oxidation wave has been previously reported by Azov et al. for TTF-containing molecular tweezers based on a 1,2,4,5-tetramethylbenzene scaffold [42–43].

**Figure 4:**
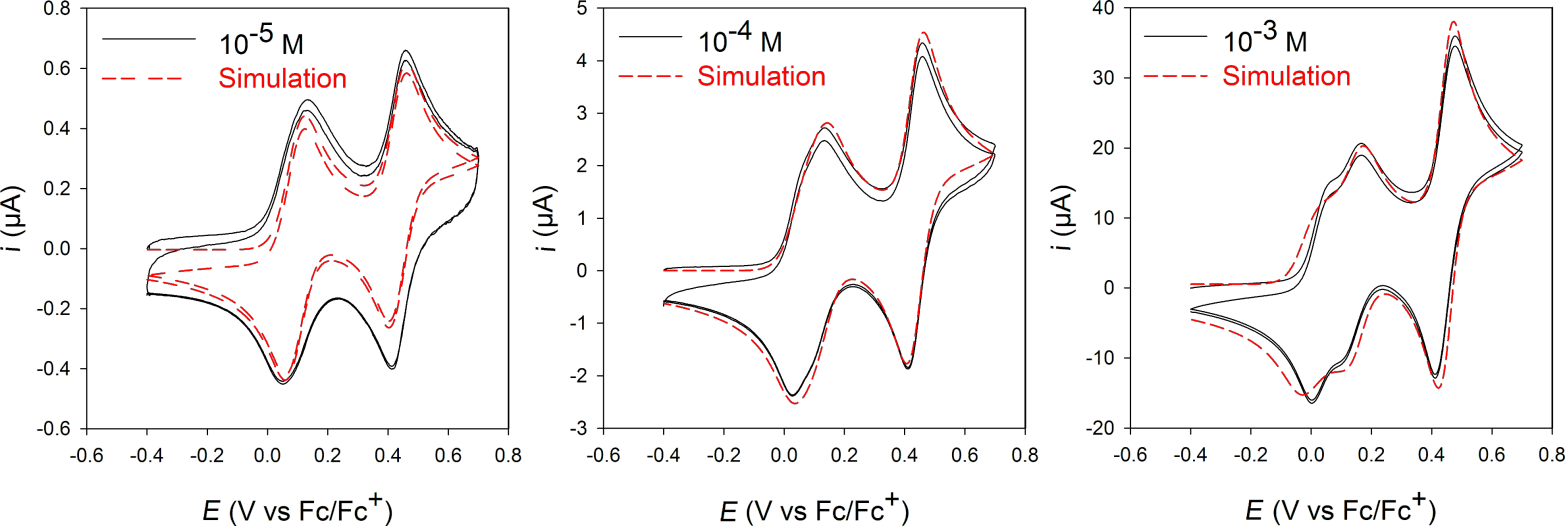
Cyclic voltammograms of molecular clip **2** at different concentrations (left: 10^−5^ M; middle: 10^−4^ M; right: 10^−3^ M) in 0.1 M TBAPF_6_/CH_2_Cl_2_/CH_3_CN 3:1 on a glassy carbon electrode at 100 mV·s^−1^. Red dashed line: electrochemical simulation performed from experiments (*E*_1_ = 0.090 V, *E*_2_ = 0.165 V, *E*_3_ = 0.435 mV, *K*_MV_ = 11400 M^−1^, *K*_DIM_ = 680 M^−1^, *k*_f_ = 1 × 10^9^ M^−1^·s^−1^ for all the forward reactions, α = 0.5 and *k*_s_ = 0.01 cm·s^−1^ for all the charge transfers).

At this stage, one should keep in mind that the distance between two TTF sidewalls within clips **2** and **3** is quite important (8.2 Å and 7.4 Å, respectively). Such a distance, even though determined in the solid state, does not seem compatible to allow an intramolecular interaction to occur between two TTF units during the first oxidation step. In order to address this issue and to explore possible intermolecular (between two clips) interactions, concentration dependence electrochemical experiments were performed. Cyclic voltammograms were recorded using molecular clip **2** at different concentrations (10^−3^ M, 10^−4^ M and 5 × 10^−5^ M) (Figure 4).

Importantly, whereas the splitting of the first oxidation wave was perfectly observed at high concentration (10^−3^ M), we noted that the phenomenon was significantly decreased at lower concentration. First, the absence of a splitting or broadening of the first oxidation process at low concentration informed us that the two TTF units are equivalent, thus excluding intramolecular interactions. Secondly, the concentration dependence and the splitting at higher concentrations provided evidence for the existence of intermolecular interactions and the formation of mixed-valence and radical cation dimer states. These results clearly demonstrated the presence of an intermolecular mixed-valence phenomenon.

**Scheme 3:**
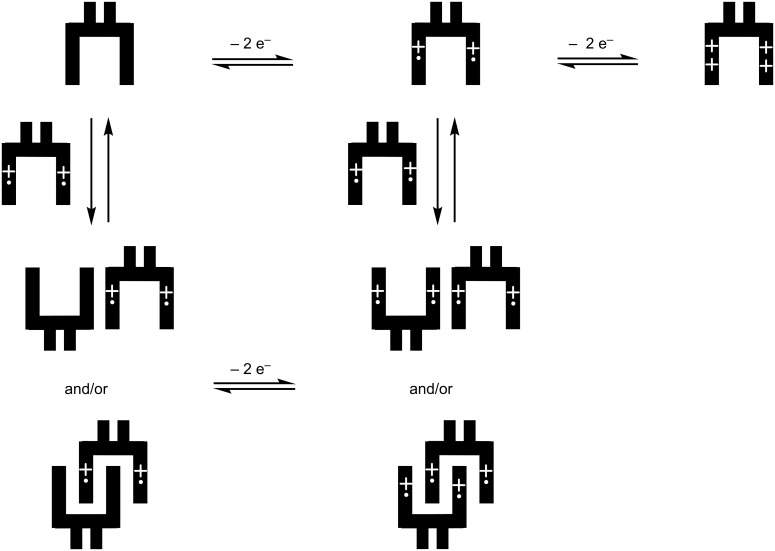
Graphical representation of the stepwise oxidation of molecular clips **1**, **2** and **3**.

According to these first results, the following model could be proposed in agreement with the successive oxidation steps in the CV of molecular clips **1**, **2** and **3** (Scheme 3). Because the two TTF units are equivalent, the first oxidation step of the clip involves a one-electron process on each TTF unit, leading to the clip bearing two radical cations. The concentration dependence shown on CVs suggested that this clip with two radical cations stack with and/or without self-assembly affording mixed-valence or π-dimer interactions. At higher potential, the tetracation is provided by another one-electron process on each TTF unit leading to a clip bearing two dications.

**Scheme 4:**
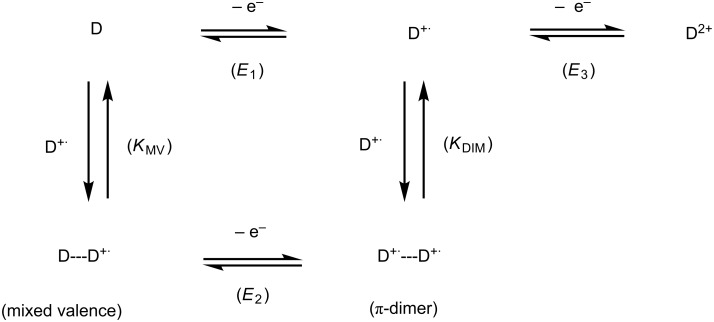
Electrochemical mechanism used to simulate the CVs of molecular clips **1**, **2** and **3**.

The graphical representation can be simulated by an electrochemical simulation program (DigiElch 7) involving an electrochemical mechanism (Scheme 4) composed by three one-electron processes and two charge-coupled chemical reactions (i.e*.,* formation of mixed-valence and dimerization via a square scheme). Due to the equivalence of the two TTF units, it is important to note that the input value of the concentration of D in Scheme 4 (D represents one TTF unit of the clip) must be twice that of the experimental concentration of a molecular clip. The good agreement between experimental data and modelled CVs over the whole range of concentration supports the graphical representation of the stepwise oxidation of molecular clips **1**, **2** and **3** (Scheme 3).

### Optical and spectroelectrochemical properties

**Figure 5:**
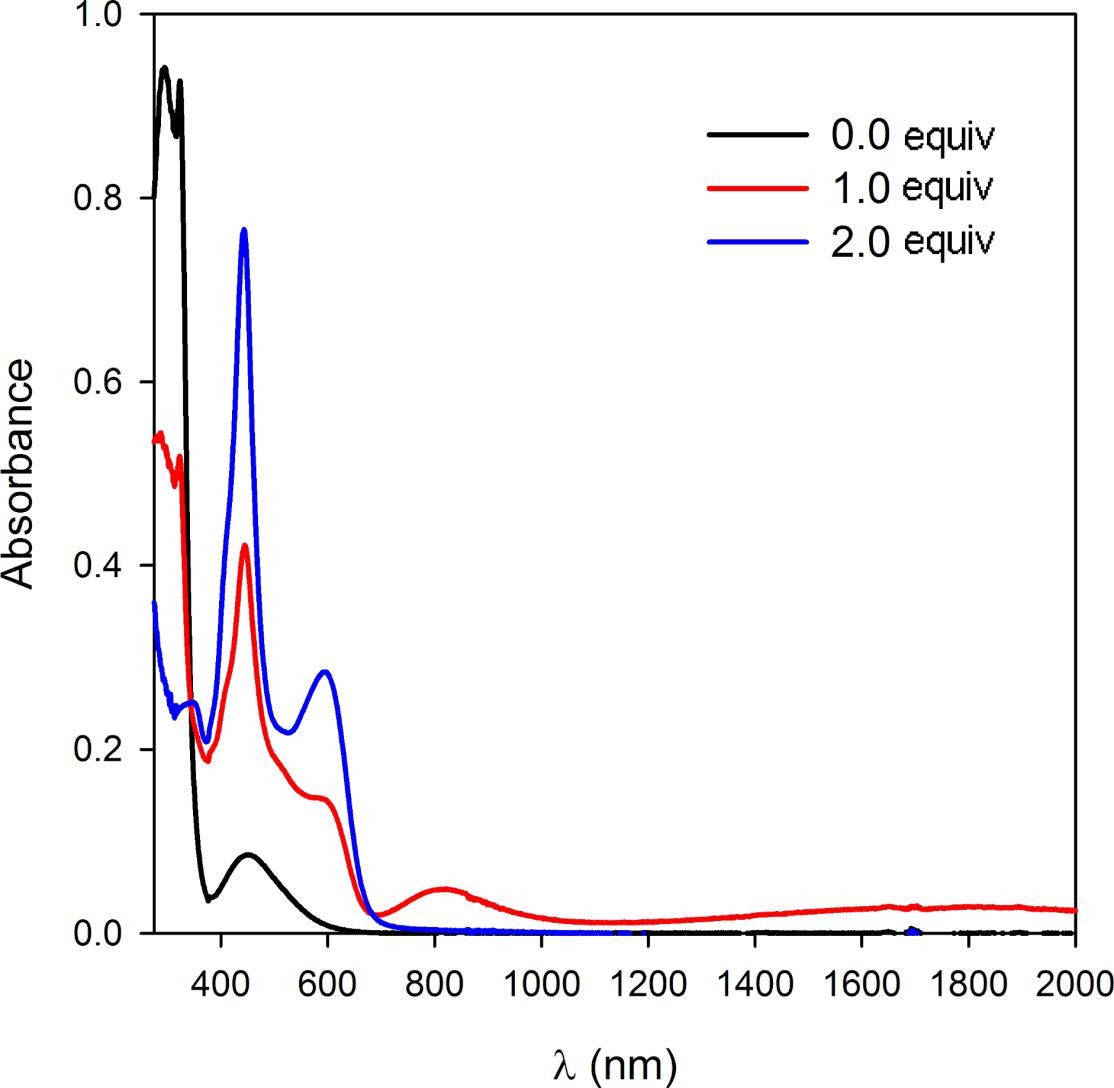
Chemical oxidation of molecular clip **1** (10^−4^ M, CH_2_Cl_2_) using aliquots of NOSbF_6_ oxidizing reagent (5 *×* 10^−3^ M, CH_3_CN).

The optical properties of oxidized states of molecular clip **1** were monitored by UV–vis–NIR spectroscopy, by successive aliquot addition of NOSbF_6_ (5 × 10^−3^ M in CH_3_CN) used as oxidizing reagent in a solution of clip **1** (10^−4^ M in CH_2_Cl_2_) in a quartz cell (0.2 cm) (Figure 5). Clip **1** bearing methoxycarbonyl groups at the periphery of the TTF framework was chosen because, to our knowledge, the optical characteristics of such a TTF derivative have not been yet reported. The redox potential (+ 0.87 V vs Fc^+^/Fc) of the NOSbF_6_ reagent is in agreement to allow the oxidation of TTF units. Chemical oxidation led to the rapid disappearance of the band at 320 nm attributed to the neutral TTF derivative and to the concomitant development of new bands characteristic for the cation radical and/or the π-dimer (440, 595 nm), bands which are characteristic for substituted TTF derivatives. After addition of nearly two equivalents of oxidizing reagent, we noted the appearance of the absorption band characteristic of the formation of the TTF dication (340 nm). As soon as we started to add aliquots of NOSbF_6_ oxidant onto molecular clip **1**, we could observe in the NIR region an absorption band centered at approximately 815 nm and a broad and weak band between 1300–2000 nm.

**Figure 6:**
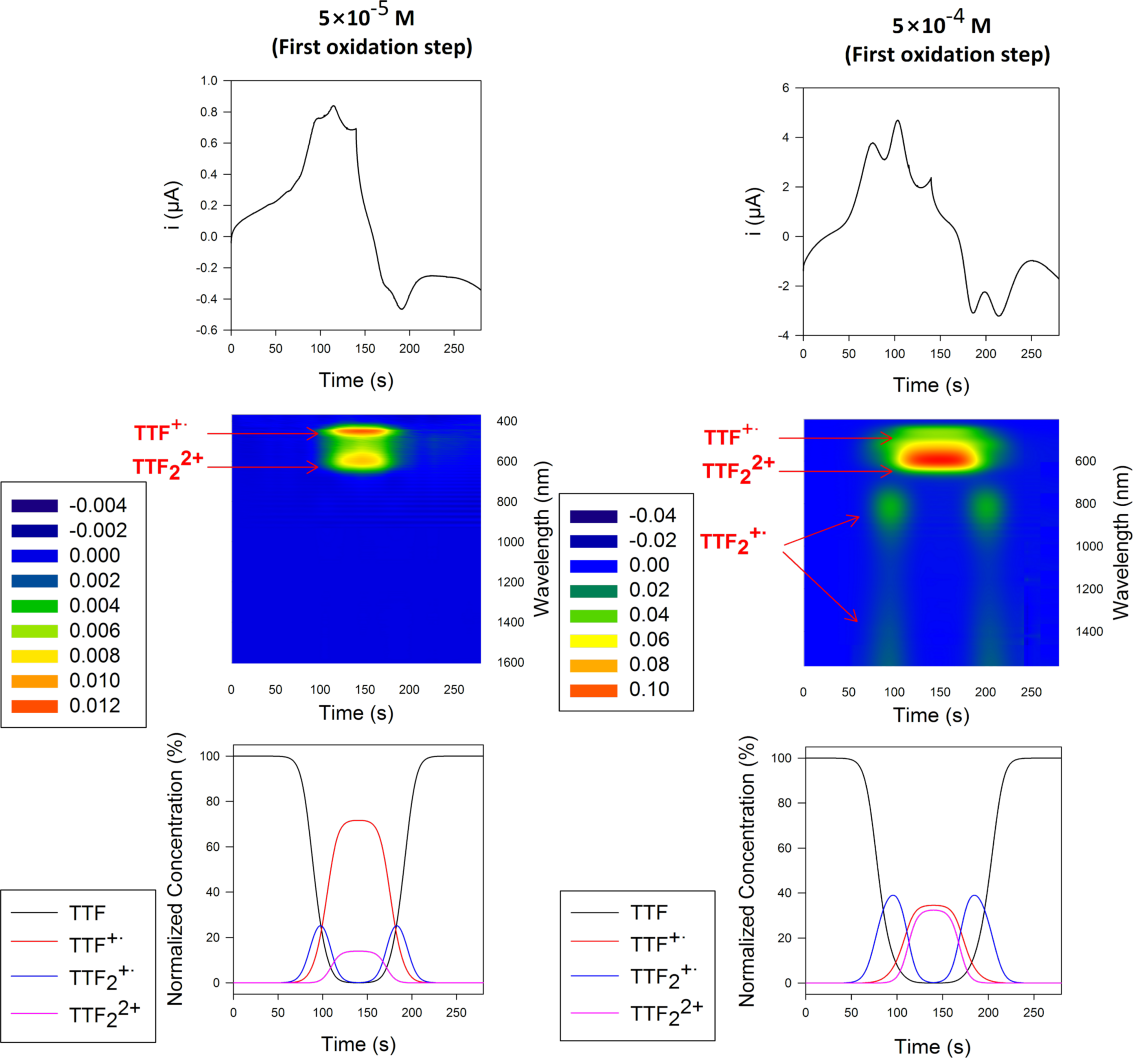
Spectroelectrochemical experiment of molecular clip **1** during the first oxidation step at different concentrations (left: 5 × 10^−5^ M; right : 5 × 10^−4^ M) in 0.1 M TBAPF_6_/CH_2_Cl_2_/CH_3_CN (3:1) on glassy carbon on Pt electrode in thin layer conditions (close to 50 µm) electrode at 5 mV·s^−1^ and 293 K. (top) CV in current vs time representation. (middle) 3D representation: *x*-axis = wavelength, *y*-axis = time and *z*-axis = absorbance. (bottom) concentration-time profiles of each simulated species in the thin layer (50 µm) calculated from electrochemical simulations of Figure 4 (*E*_1_ = 0.090 V, *E*_2_ = 0.165 V, *E*_3_ = 0.435 mV, *K*_MV_ = 11400 M^−1^, *K*_DIM_ = 680 M^−1^, *k*_f_ = 1 × 10^9^ M^−1^·s^−1^ for all the forward reactions, α = 0.5 and *k*_s_ = 0.01 cm·s^−1^ for all the charge transfers).

Known to be a powerful tool for analyzing the formation of π-dimers (DIM) and mixed-valence (MV) systems, time resolved spectroelectrochemical experiments [44–46] were performed on molecular clip **1** in order to probe the first oxidation step at two different concentrations between 350 and 1700 nm in 0.1 M TBAPF_6_/CH_2_Cl_2_/CH_3_CN (3:1). At 5 × 10^−5^ M, only two absorption bands (i.e., 450 and 600 nm) were observed (Figure 6). Confirming a concentration dependence, the absorbance profile at 5 × 10^−4^ M was different from the one obtained at 5 × 10^−5^ M. Two additional absorption bands simultaneously emerged at the beginning of the oxidation process and the end of the reduction process: one at 825 nm and a broad band in near infrared range beyond 1300 nm (Figure 6).

In agreement with the simulated concentration–time profiles in thin layer conditions (Figure 6 – bottom), the appearance of the absorption bands centered around 825 and beyond 1300 nm were attributed to the formation of [(TTF)_2_]^+^ mixed-valence dimer [47–48] and the absorption bands centered around 600 nm to the (TTF˙^+^)_2_ π-dimer [49]. The band close to 825 nm could be assigned to cation radicals stack with self-assembly affording mixed-valence. Nevertheless, at this stage, it would be premature to conclude with certainty. The origin of this band is currently under investigation and requires complementary studies to carry out on different substituted molecular clips.

By analogy with unsubstituted TTF derivative, these three bands reasonably support the presence of mixed-valence and cation radical dimers during the oxidation process. Whereas the phenomenon of dimer formation of the TTF cation radical is commonly observed in the solid state, it was also described in solution at low temperature in a concentrated TTF solution [50], or at room temperature in a dilute solution. In the latter case, the characterization of [(TTF)_2_]^+^˙ mixed-valence dimer and/or (TTF^+^˙)_2_ dimer species concerned systems for which the dimer stabilization is resulting from the close proximity of the two TTF units. That is the case for conjugated bisTTF systems [51], bisTTF-substituted calix[4]arenes [52], or supramolecular architectures such as [3]catenane [53], cucubit[8]uril [54] or self-assembled cages [55] which facilitate the formation of TTF dimers.

**Figure 7:**
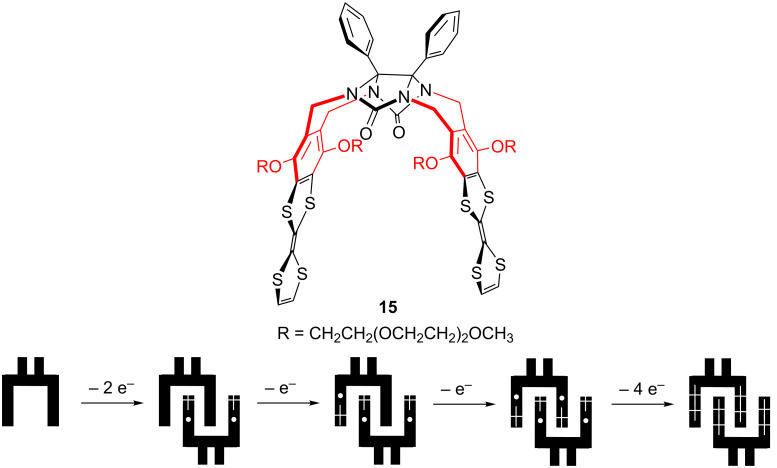
Molecular structure of molecular clip **15** and representation of its stepwise oxidation processes proposed by Chiu et al. [58].

Studying the glycoluril-based molecular clip **15** presenting also TTF sidewalls [56–57], Chiu et al. have observed the mixed-valence and radical cation dimer states at high concentration (10^−3^ M) and at room temperature [58]. Indeed, the CV of molecular clip **15** exhibited four consecutive oxidation waves, which were interpreted by a succession of oxidation processes of the TTF sidewalls (Figure 7). These dimer interactions were predicted to exist at room temperature by theoretical investigation realized on this system [59].

**Figure 8:**
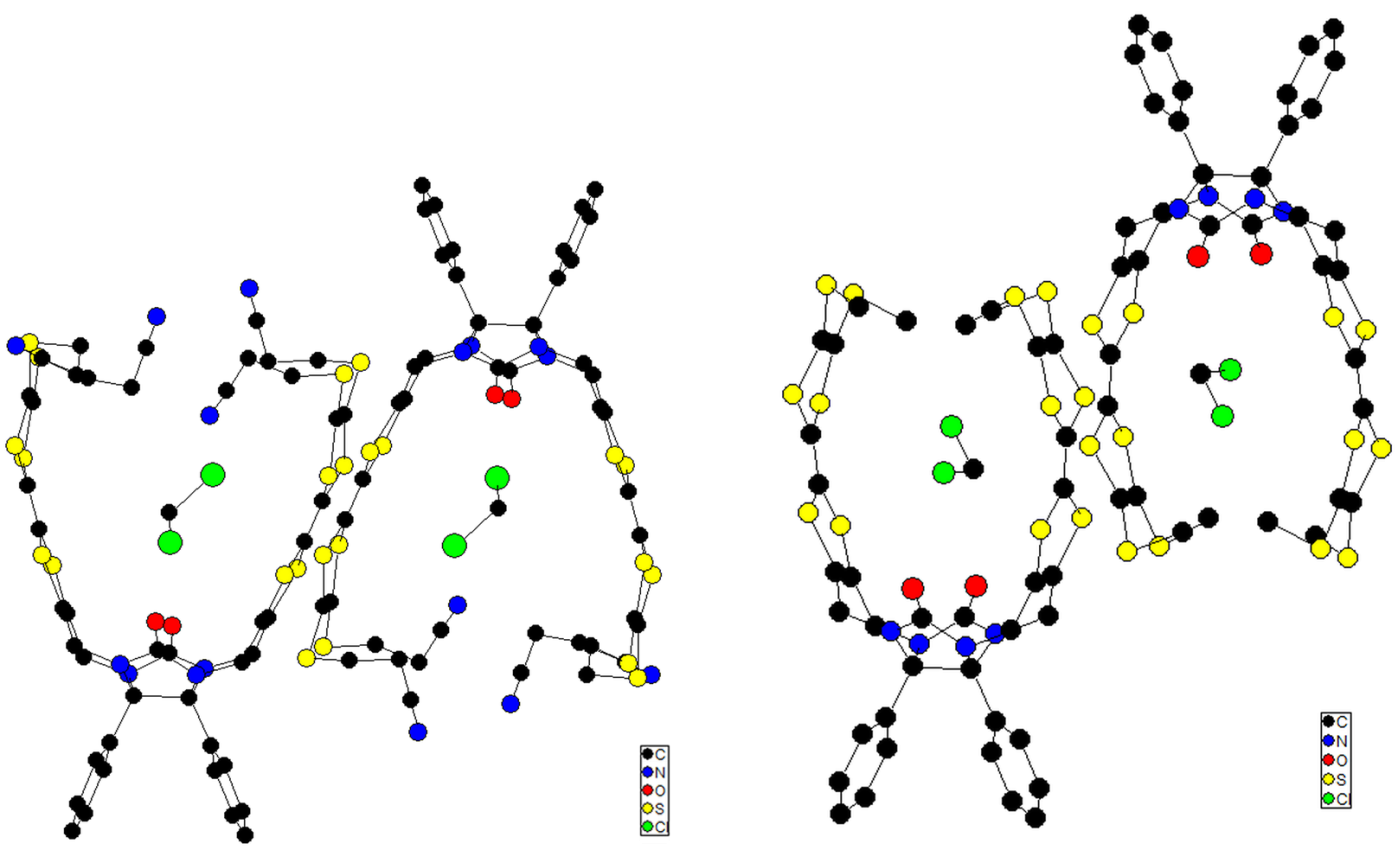
Molecular packing diagram of clips **2** (left) and **3** (right) obtained from X-ray analysis. A molecule of CH_2_Cl_2_ is included inside the cavity of each clip. Hydrogen atoms are omitted for clarity.

By comparison with these results observed for molecular clip **15** [58], it is clear that clips **1–3** exhibit [(TTF)_2_]^+^˙ mixed-valence dimer and/or (TTF^+^˙)_2_ dimer species but their self-association organization could not be yet demonstrated. The presence of more or less sterically bulky groups at the periphery of the TTF moieties does not provide an advantage for the self-assembly of dimers. Moreover, this dimerization phenomenon which is driven by the inclusion of one sidewall into the cavity of the opposing clip is not observed in the solid state for clips **2** and **3** (Figure 8). This typical packing arrangement was previously observed in many cases of glycoluril-based molecular clips containing two aromatic sidewalls [26,60–63]. On the contrary, considering the unit cell for single crystals of clip **2**, X-ray analysis showed that two neighboring molecules of clip **2** appeared in a head-to-tail arrangement and short TTF···TTF intermolecular distances (3.51 Å) were determined between two clips in the solid state.

### Binding properties

In order to establish the influence of electronic and spatial properties of the clips towards binding ability of neutral electrodeficient guest molecules, we have chosen compounds **3** and **4** for this study. Molecular clip **3** presents better π-donating ability according to CV study and a smaller interplanar TTF distance (7.41 Å compared to calculated 9.2 Å for clip **4**). Binding properties were studied using 1,3-dinitrobenzene (*m*-DNB) as a weak and small aromatic electron acceptor molecule. It should be noted that Nolte et al. have successfully observed the complexation of *m*-DNB using a glycoluril-based receptor bearing 2,7-dimethoxynaphthalene walls (*K*_a_ = (115 ± 10) M^−1^ ) [64]. In the latter case, it was proposed that binding was occurring through an induced-fit mechanism with a recognition process on the basis of the size rather than the acceptor strength. We can suppose that the presence of stronger π-donor TTF sidewalls in clip **3** favorize donor–acceptor interactions and consequently the binding properties towards *m*-DNB. We have also checked the influence of the redox properties towards binding ability by studying the strong electrodeficient acceptor F_4_-TCNQ.

#### Interaction with 1,3-dinitrobenzene (*m*-DNB)

**Figure 9:**
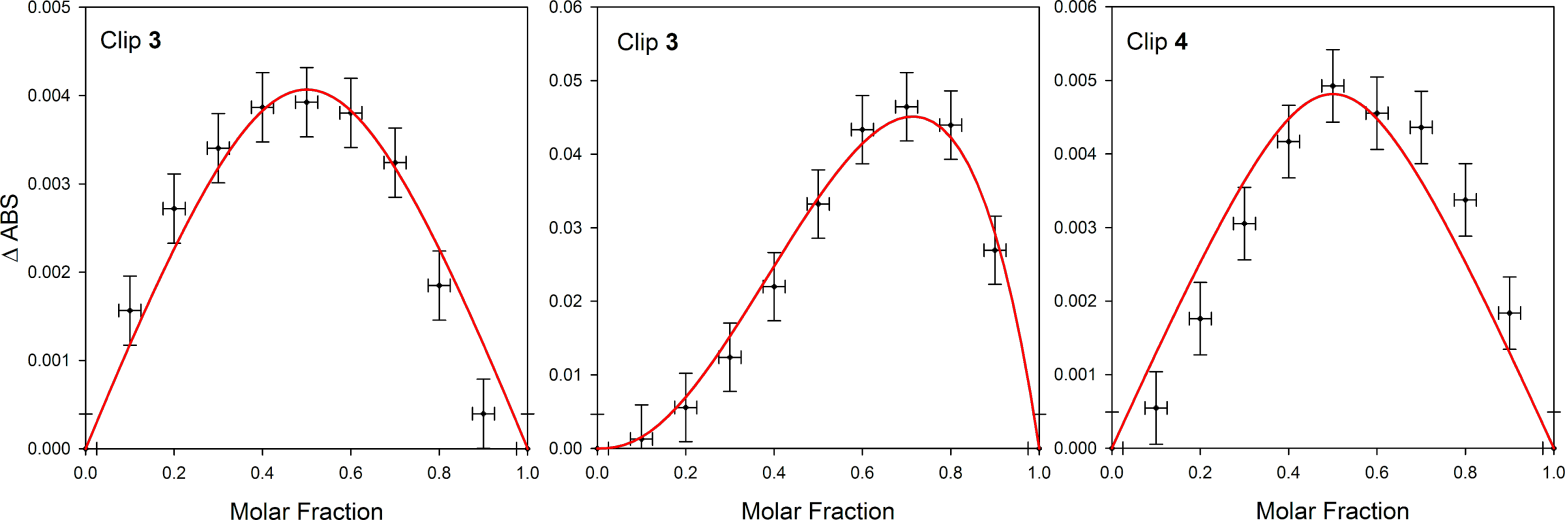
Left: Job plot analysis for DNB vs molecular clip **3** ([**3** + DNB] = 10^−3^ M in *o*-C_6_H_4_Cl_2_ at 800 nm) at room temperature. Middle: Job plot for F_4_-TCNQ vs molecular clip **3** at room temperature ([**3** + F_4_-TCNQ] = 10^−5^ M in CH_2_Cl_2_ at 860 nm) consistent with a 2:1 binding stoichiometry. Right: Job plot for F_4_-TCNQ vs molecular clip **4** at room temperature ([**4** + F_4_-TCNQ] = 10^−5^ M in CH_2_Cl_2_ at 860 nm) in agreement with a 1:1 binding stoichiometry.

The host–guest affinity was detected by UV–visible spectroscopy upon titration of clip **3** (10^−3^ M in *o*-C_6_H_4_Cl_2_) with addition of *m*-DNB (10^−1^ M in *o*-C_6_H_4_Cl_2_) aliquots. No additional change of the spectra was observed after the addition of one equivalent of *m-*DNB (−1.30 V vs Fc^+^/Fc) [65] which is in agreement with the formation of a 1:1 complex. A Job plot carried out in *o*-C_6_H_4_Cl_2_ between clip **3** and *m-*DNB shows a maximum at 0.5, confirming the formation of the 1:1 complex (*m*-DNB@clip **3**) (Figure 9 left). The association constant was determined to be *K*_a_ = (7 ± 3) × 10^3^ M^−1^ by exploiting the Job plot analysis according to literature [66]. Despite many efforts devoted to the search for a complexation of *m*-DNB using molecular clip **4**, we could not observe any host–guest binding interaction. These results suggest that electronic and spatial properties of the cavity constitute fundamental parameters for binding the *m*-DNB guest. The binding of such a weak electron acceptor was successful for molecular clip **3** presenting the most suitable intramolecular distance between TTF sidewalls and the strongest π-donor ability.

#### Interaction with tetrafluoroquinodimethane (F_4_-TCNQ)

**Figure 10:**
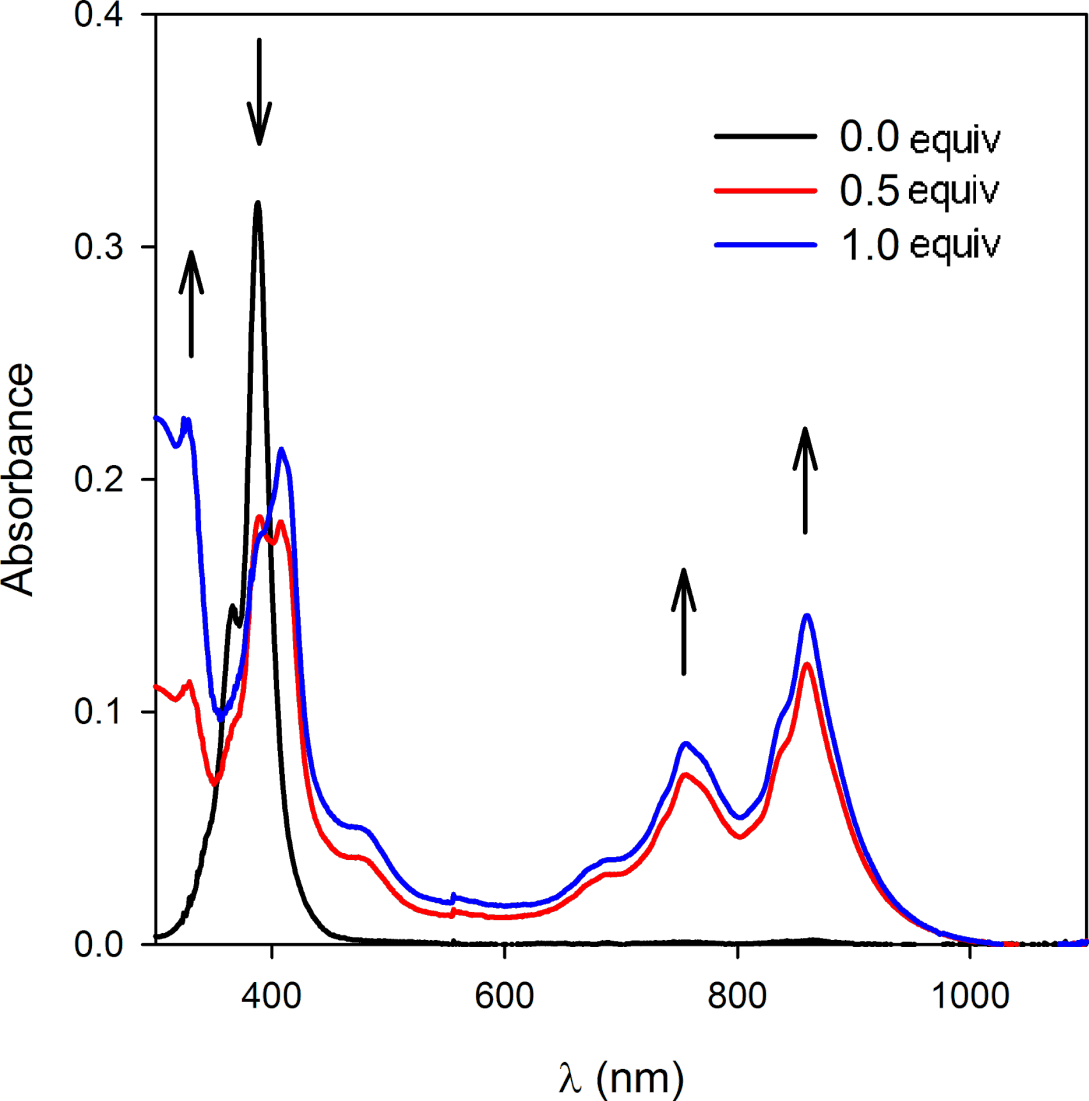
UV–visible absorption spectra of F_4_-TCNQ (CH_2_Cl_2_, 10^−5^ M) upon titration with molecular clip **3** (CH_2_Cl_2_, 5 × 10^−4^ M).

The binding affinity of molecular clip **3** (5 × 10^−4^ M in CH_2_Cl_2_) was studied by a UV–visible titration with the electron acceptor F_4_-TCNQ (10^−5^ M in CH_2_Cl_2_). Addition of aliquots of molecular clip **3** onto a F_4_-TCNQ solution showed the presence of an isosbestic point at 395 nm with the concomittent formation and increase of bands at 760 and 860 nm which could be attributed to the progressive formation of the F_4_-TCNQ radical anion (Figure 10) [67–68]. The increase of the bands around 750 and 850 nm was attributed to the cumulative contribution of the increasing formation of TTF cation radical species and the F_4_-TCNQ anion radical.

A Job plot carried out in CH_2_Cl_2_ between clip **3** and F_4_-TCNQ exhibited a maximum at around 0.7, a finding that corroborates with the formation of a 1:2 complex (F_4_-TCNQ)_2_@clip **3** (Figure 9 middle) with an average binding constant of *K*_a_ = (8 ± 5) × 10^8^ M^−1^.

Similar studies were carried out using molecular clip **4** and quantitative measurements were performed using UV–visible titration considering the changes in the spectra of F_4_-TCNQ at 860 nm upon addition of sensor **4**. The construction of the Job plot is in agreement with the 1:1 binding stoichiometry between clip **4** and F_4_-TCNQ with a maximum centered at a molar ratio of 0.5 (Figure 9 right). The high association constant *K*_a_ = (1.3 ± 0.8) × 10^6^ M^−1^ in CH_2_Cl_2_ confirms that this rigidified molecular clip **4** is an efficient receptor for F_4_-TCNQ guest electron acceptor.

**Figure 11:**
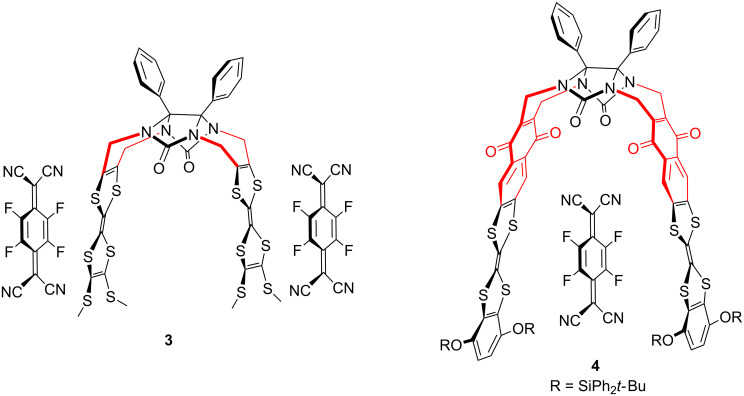
Redox interaction (left) and complexation (right) of F_4_-TCNQ with molecular clips **3** and **4**.

This difference in binding interaction between host molecular clips **3** and **4** towards the F_4_-TCNQ guest could be explained by their electrochemical properties. By comparing the first oxidation potential of clips **3** and **4** with the first reduction potential of F_4_-TCNQ (*E*_app_^0^_red1_ = + 0.14 V vs Fc^+^/Fc – Figure 3), it is clear that F_4_-TCNQ guest presents a high reduction potential, sufficient to allow the oxidation of molecular clip **3**. Consequently, the (F_4_-TCNQ)_2_@clip **3** complex should result from a redox interaction between the host and the guest. On the other hand, the oxidation potential of molecular clip **4** is increased due to the introduction of the naphthoquinone spacer. Consequently, the corresponding 1:1 stoichiometry should reasonably correspond to the binding of F_4_-TCNQ inside the cavity of clip **4** (Figure 11). This binding of F_4_-TCNQ also results from favourable spatial parameters with the increase of the intramolecular distance between the two TTF sidewalls. Thanks to the good π-donor ability of the TTF sidewalls, in combination with a well-suited size of the cavity, it is here demonstrated that molecular clip **4** constitutes one of the rare examples of systems exhibiting good affinity for sandwiching F_4_-TCNQ as an electron poor guest.

## Conclusion

We have presented original synthetic strategies leading to glycoluril-based molecular clips containing electroactive TTF sidewalls. In particular, the one-step preparation of molecular clip using the N-tetraalkylation of glycoluril constitutes a powerful versatile method allowing an easy access to new architectures for which electrochemical properties can be tuned by simple modification of peripheral substituents on the TTF moiety. Cyclic voltammetry and spectroelectrochemical measurements demonstrated that the mixed-valence state in these fused glycoluril-TTF molecular clips seems to originate from intermolecular TTF interactions, according to measurements at various concentrations and to cyclic voltammogram simulations. Spatial and electrochemical properties were shown to be fundamental parameters towards the binding interaction of a weak (*m*-DNB) or strong (F_4_-TCNQ) electrodeficient guest. Consequently, the selectivity for a given target guest could be precisely tuned via the choice of the molecular clip according to its electrochemical and spatial considerations.

## Experimental

### Synthesis

Molecular clips **1–3** [18] and **4** [19] were synthesized according to our previous reports.

### Electrochemical and spectroelectrochemical experiments

Electrochemistry and time-resolved spectroelectrochemistry in solution were performed using the already described home self-made cell [44–45]. Electrochemical measurements were carried out using a platinum wire counter electrode and a silver wire as a quasi-reference electrode with a Biologic SP-150 potentiostat driven by the EC-Lab software including ohmic drop compensation. Experiments were recorded in dry HPLC-grade acetonitrile and dichloromethane with tetrabutylammonium hexafluorophosphate (Bu_4_NPF_6_, electrochemical grade, Fluka) as supporting electrolyte. All solutions were prepared and transferred into the spectroelectrochemical cell in a glove box containing dry, oxygen-free (<1 ppm) argon, at room temperature.

Spectrophotometric measurements were carried out in direct reflexing mode on the working electrode (i.e., Pt or glassy carbon) with a homemade bench composed of different Princeton Instruments modules (light sources, fibers, monochromators, spectroscopy camera and software). The connection between the light source, the cell and the spectrophotometer is ensured through a “Y-shaped” optical fiber bundle: 18 fibers guide the light to the cell, and 19 fibers collect the reflected light from the cell to visible (320–1080 nm / maximum acquisition frequency 2 MHz) and IR (900–1700 nm / maximum acquisition frequency 8 MHz) CCD detectors. The sensitivity of the spectroscopic measurement (<3 electrons at 100 kHz and <13 electrons at 2 MHz between 320 and 1080 nm; 400 electrons (high gain) and 5000 electrons (low gain) between 900 nm and 1700 nm) allows performing a spectroelectrochemistry experiment under the usual conditions of electrochemistry.
